# Endoplasmic reticulum stress-regulated CXCR3 pathway mediates inflammation and neuronal injury in acute glaucoma

**DOI:** 10.1038/cddis.2015.281

**Published:** 2015-10-08

**Authors:** Y Ha, H Liu, Z Xu, H Yokota, S P Narayanan, T Lemtalsi, S B Smith, R W Caldwell, R B Caldwell, W Zhang

**Affiliations:** 1 Department of Ophthalmology and Visual Sciences, The University of Texas Medical Branch, Galveston, TX, USA; 2 Center for Biomedical Engineering, The University of Texas Medical Branch, Galveston, TX, USA; 3 Vascular Biology Center, Georgia Regents University, Augusta, GA, USA; 4 Department of Ophthalmology, Asahikawa Medical University, Asahikawa, Japan; 5 College of Allied Health Sciences, Georgia Regents University, Augusta, GA, USA; 6 Cellular Biology and Anatomy, Georgia Regents University, Augusta, GA, USA; 7 Department of pharmacology and Toxicology, Georgia Regents University, Augusta, GA, USA; 8 VA Medical Center, Augusta, GA, USA; 9 Neuroscience and Cell Biology, The University of Texas Medical Branch, Galveston, TX, USA

## Abstract

Acute glaucoma is a leading cause of irreversible blindness in East Asia. The mechanisms underlying retinal neuronal injury induced by a sudden rise in intraocular pressure (IOP) remain obscure. Here we demonstrate that the activation of CXCL10/CXCR3 axis, which mediates the recruitment and activation of inflammatory cells, has a critical role in a mouse model of acute glaucoma. The mRNA and protein expression levels of CXCL10 and CXCR3 were significantly increased after IOP-induced retinal ischemia. Blockade of the CXCR3 pathway by deleting CXCR3 gene significantly attenuated ischemic injury-induced upregulation of inflammatory molecules (interleukin-1*β* and E-selectin), inhibited the recruitment of microglia/monocyte to the superficial retina, reduced peroxynitrite formation, and prevented the loss of neurons within the ganglion cell layer. In contrast, intravitreal delivery of CXCL10 increased leukocyte recruitment and retinal cell apoptosis. Inhibition of endoplasmic reticulum (ER) stress with chemical chaperones partially blocked ischemic injury-induced CXCL10 upregulation, whereas induction of ER stress with tunicamycin enhanced CXCL10 expression in retina and primary retinal ganglion cells. Interestingly, deleting CXCR3 attenuated ER stress-induced retinal cell death. In conclusion, these results indicate that ER stress-medicated activation of CXCL10/CXCR3 pathway has an important role in retinal inflammation and neuronal injury after high IOP-induced ischemia.

Acute glaucoma is the major form of glaucoma in East Asia where it is a leading cause of irreversible blindness.^1^ In Western countries, it is less common, but it still has much higher rate to induce vision impairment and blindness than open-angle glaucoma.^2^ Acute glaucoma is a medical emergent condition when intraocular pressure (IOP) is suddenly increased because of blocked drainage canals.^1, 2^ Prompt treatment is needed to avoid irreversible glaucomatous optic nerve damage.^1^ Nevertheless, in a substantial portion of patients, acute glaucoma continues progressing to blindness in spite of intensive medical treatment.^3^ A rapid rise in IOP that exceeds retinal perfusion pressure is known to cause retinal ischemia and induce retinal neuronal cell death.^2, 4^ However, the mechanisms by which elevated IOP induces retinal neuronal injury in acute glaucoma are largely unknown.

Inflammation is the body's defense system against pathogens,^5^ whereas excessive or uncontrolled inflammation induces tissue injury and results in diseases. In the central nervous system (CNS), inflammation has been recognized as a key player in many neurodegenerative diseases, such as Alzheimer's disease, Parkinson's disease, and Huntington's disease.^6^ Inflammation is involved in the development of glaucoma given that the levels of inflammatory cytokines (e.g., TNF-*α*, interleukin-6, CCL2) and adhesion molecules (e.g., p-selectin) are increased in retina during glaucoma.^7, 8^ Moreover, in an animal model of acute glaucoma, inflammatory pathways including toll-like receptors and inflammasome are activated and contribute to retinal neuronal injury.^4, 9^ Nonetheless, the key mediators that control inflammatory cell recruitment and activation in this process remain to be elucidated.

Chemokines are a family of pro-inflammatory peptides (8-15 kD) that are produced locally in tissues and mediate leukocyte and microglia directional migration and activation during inflammatory reactions by binding to specific chemokine receptors in the membrane of their respective target cells. CXCR3 is the common receptor for three chemokines belonging to the CXC subclass, namely CXCL9, CXCL10, and CXCL11,^10^ whereas it binds to CXCL4 with low affinity.^11, 12, 13^ CXCR3 is critical to mediate the recruitment of activated T cells and microglia/macrophage.^10, 14, 15, 16, 17^ CXCL10 expression is increased in the central nervous system in neurodegenerative diseases including Alzheimer's disease and multiple sclerosis.^18, 19, 20, 21^ The activation of CXCL10/CXCR3 axis has also been shown to promote microglia recruitment and induce neuronal cell death in several models of neurodegeneration.^22, 23^ The role of CXCL10/CXCR3 pathway in glaucomatous optic neuropathy is unknown.

In this study, we demonstrate that the CXCL10/CXCR3 axis is activated in a mouse model of acute glaucoma and the activation of this pathway is essential for retinal inflammation and neuronal injury. Moreover, the upregulation of CXCL10 and CXCR3 expression in retina is at least partially mediated by endoplasmic reticulum (ER) stress after ischemia.

## Results

### CXCL10 and CXCR3 are upregulated after IOP-induced retinal ischemia

**Figure 1:**
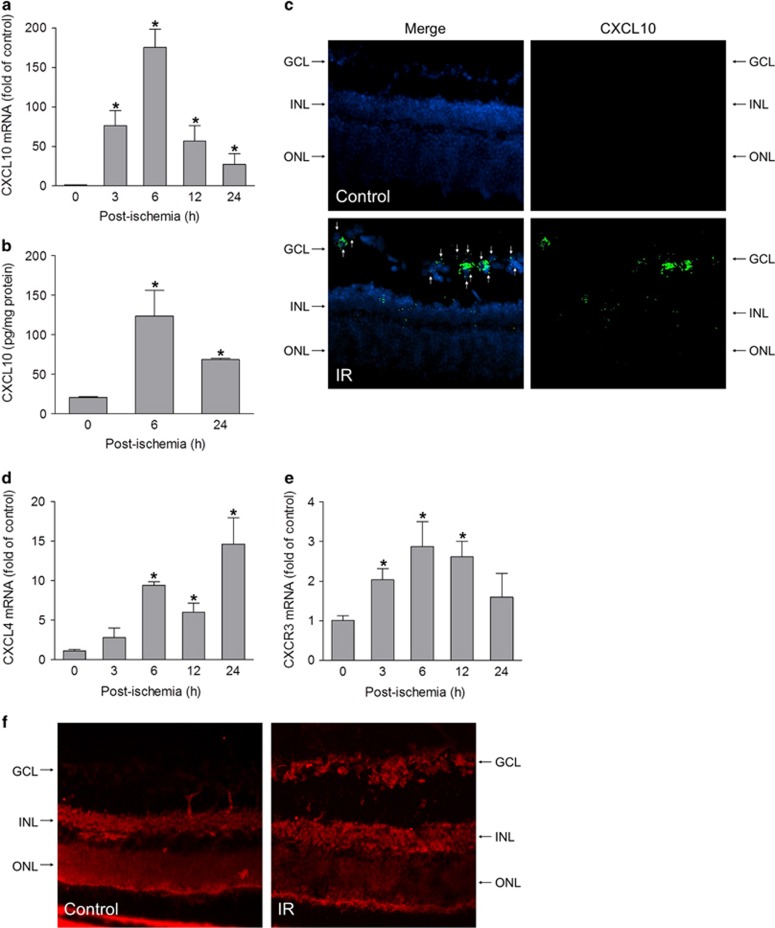
Ischemia-reperfusion induces the expression of CXCR3 and its ligands in retina. Ischemia-reperfusion (IR) was induced in wild-type (WT) mice and retinas were collected at 3, 6, 12, and 24 h after IR. (**a**) CXCL10 mRNA was determined by quantitative RT-PCR (qPCR). (**b**) CXCL10 protein expression was analyzed by ELISA. (**c**) *In situ* hybridization of CXCL10 mRNA. Retinal frozen sections from control and IR-performed mice at 6 h after IR were hybridized with a probe against mouse CXCL10 and detected with RNAscope Fluorescent Multiplex Kit. Green fluorescent signal reflects CXCL10 mRNA expression and DAPI (blue) stains nuclei. Arrows indicate CXCL10-expressed retinal ganglion cells. GCL: ganglion cell layer; INL: inner nuclear layer; ONL: outer nuclear layer. (**d** and **e**) The mRNA levels of CXCL4 and CXCR3 were determined by qPCR. (**f**) Retinal frozen sections from control and IR-performed mice at 6 h after IR were incubated with an antibody against CXCR3. Fluorescent signal (red) reflects CXCR3 staining. *P<0.05 compared with control. *N*=3–4

To investigate whether CXCR3 signaling is implicated in acute glaucoma, we examined the expression of CXCR3 and its ligands in a mouse model of acute glaucoma, in which retinal ischemia is induced by acute elevation of IOP.^4^ Analysis of mRNA by quantitative PCR revealed that CXCL10 expression was markedly increased in retinal tissue and reached a peak at 6 h after ischemia (175-fold of control) (Figure 1a). Consistent with the increase in mRNA, CXCL10 protein was significantly increased as determined by enzyme-linked immunosorbent assay (ELISA) (Figure 1b). CXCL10 mRNA-positive cells were distributed in different retinal layers including cells in the ganglion cell layer (GCL) (Figure 1c). The expression of CXCL4, which binds with low affinity to CXCR3, was elevated after retinal ischemia though expressed later than CXCL10 (Figure 1d). In contrast, the expression of two other ligands, CXCL9, and CXCL11, was undetectable. CXCR3 mRNA was also rapidly upregulated after retinal ischemia (Figure 1e). Immunolocalization analysis showed that immunoreactivity for CXCR3 was present in the inner nuclear layer (INL) and outer nuclear layer in non-injured retina. After ischemic injury, the immunoreactivity for CXCR3 was robustly increased in cells in the GCL (Figure 1f). These data suggest that the CXCL10/CXCR3 axis may have a role in the pathogenesis of acute glaucoma.

### CXCR3 mediates retinal inflammation after ischemic injury

**Figure 2:**
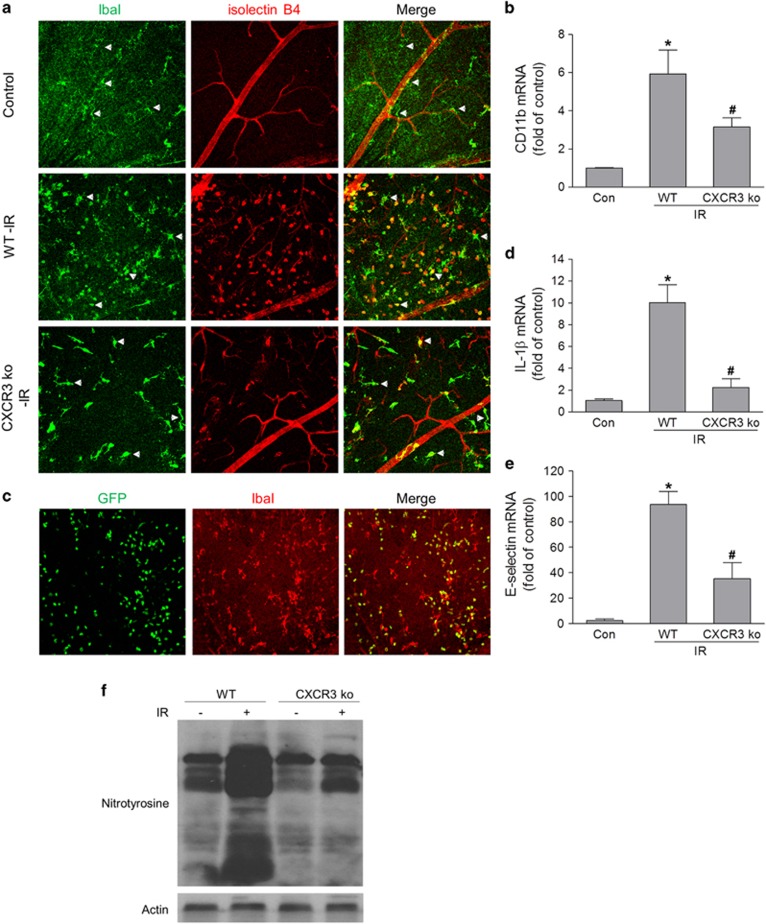
CXCR3 deletion reduces retinal inflammation as well as oxidative and nitrosative stress in retina after IR. Ischemia-reperfusion was induced in WT and CXCR3 ko mice and retinas were collected at 24 h after IR. (**a**) Retina was stained with isolectin B4 (red, for vessels and activated microglia/monocyte) and IbaI antibody (green, for microglia/monocyte). Arrows show microglia/monocyte with different morphology suggesting different activation status. (**b**) The mRNA level of CD11b in retinas was analyzed by qPCR. (**c**) WT mice received bone marrow transplant from GFP mice and 6 weeks later they were subjected to IR. Retinas were fixed and labeled for IbaI (red) at 24 h after IR. (**d** and **e**) qPCR analysis of IL-1*β* and E-selectin mRNA expression in retina at 24 h after IR. (**f**) Nitrotyrosine level in retina was analyzed by western blot at 24 h after IR. Actin was used as an internal loading control. *P<0.05 compared with control; ^#^*P*<0.05 compared with WT-IR. *N*=3–4

As CXCR3 is expressed in microglia/monocytes,^22, 24^ we explored the action of microglia/monocytes after ischemic injury. The distribution of microglia/monocyte, as determined by staining for their specific marker IbaI, revealed a prominent increase in the number of IbaI-positive cells in the superficial retina of wild-type (WT) mice after retinal ischemia (Figure 2a) compared with controls. In addition, the IbaI-positive cells were ramified in control retina, whereas many of them in the ischemia-injured retina had phagocytic (ameboid) morphology and were isolectin B4 positive, indicating an activated phenotype.^25^ In contrast, IbaI- and isolectin B4-positive cells were reduced in ischemic retinas of CXCR3-deficient mice and these cells exhibit a significantly less-activated phenotype. In accordance with increased microglia/monocyte recruitment and activation, the expression of CD11b, a marker for microglia/monocyte activation, was also remarkably increased in the retina after ischemic injury, whereas deleting CXCR3 attenuated its upregulation (Figure 2b).

To determine whether IbaI-positive cells were derived from retinal local microglia or from monocytes in circulation, we transplanted bone marrow of green fluorescent protein (GFP) mice to WT mice. Our data showed that many IbaI-positive cells were monocytes from the circulation (GFP positive) and some IbaI-positive cells were retinal microglia (GFP negative) (Figure 2c), indicating that both microglia and monocytes were recruited to the superficial retina after ischemic injury.

As interleukin-1*β*, a major product of inflammasome, has an important role in inflammation and neurotoxicity in the central nervous system and acute glaucoma,^4^ and E-selectin is involved in the process of leukocyte recruitment by mediating its initial attachment and rolling,^26^ we further determined their expression in ischemia-injured retina. We found that there were significant increases in interleukin-1*β* and E-selectin in WT retinas after ischemic injury, which were markedly blocked by deleting CXCR3 (Figures 2d and e). Together, these data indicate that the activation of CXCR3 pathway mediates microglia/monocyte recruitment and activation and retinal inflammatory reactions after IOP-induced retinal ischemia.

### CXCR3 is critically involved in oxidative and nitrosative stress after ischemic injury

During inflammation, local retinal cells and/or recruited leukocytes produce superoxide and nitric oxide, which can not only kill pathogens but also induce tissue injury. To determine whether the activation of CXCR3 is involved in oxidative and nitrosative stress after retinal ischemia, we examined the formation of peroxynitrite in retinal lysates. Peroxynitrite is rapidly formed through the reaction of superoxide and nitric oxide and is an indicator for oxidative and nitrosative stress. Western blot analysis of nitrotyrosine, a marker of peroxynitrite, revealed a prominent increase of peroxynitrite formation in WT retina after ischemic injury. However, this increase was blocked by CXCR3 deletion (Figure 2f). This result suggests that CXCR3 pathway is involved in retinal oxidative and nitrosative stress after IOP-induced retinal ischemia.

### CXCR3 pathway has a predominant role in IOP-induced retinal neuronal cell damage

**Figure 3:**
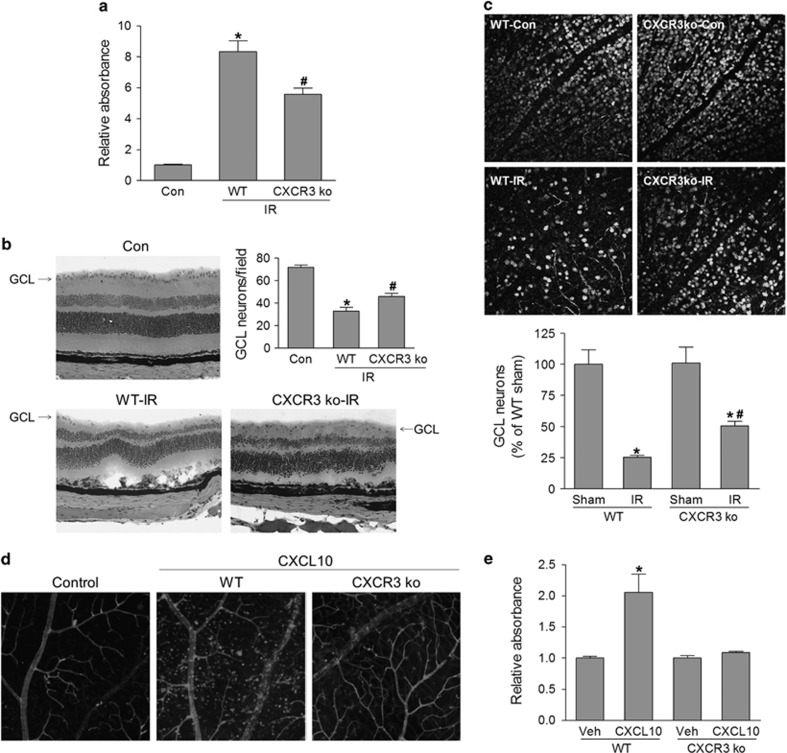
CXCR3 is necessary and sufficient for IR-induced GCL neuronal cell loss. WT and CXCR3 ko mice were subjected to IR. (**a**) Retinas were collected at 24 h after IR and analyzed for histone-associated DNA fragmentation to determine apoptosis. (**b**) Hematoxylin & eosin (H&E) staining of retinal section at 7 days after IR. The number of cells in ganglion cell layer (GCL) was quantified. (**c**) Retinas were immunostained with NeuN antibody at 7 days after IR and images were taken by confocal microscopy. NeuN-positive cells in GCL were quantified and normalized to uninjured controls. *P<0.05 compared with sham control; ^#^*P*<0.05 compared with WT-IR. (**d** and **e**) WT and CXCR3 ko mice were intravitreally injected with CXCL10 (1 μg/eye) or vehicle (PBS) and retinas were collected after 24 h from injection. (**d**) Retinas were stained with isolectin B4 to view vessels and recruited microglia/monocyte. (**e**) Retinal cell apoptosis was analyzed by detecting histone-associated DNA fragmentation. *P<0.05 compared with vehicle- treated WT or CXCL10-treated CXCR3 ko mice. *N*=3–4

The loss of retinal neurons in the GCL is a hallmark of glaucoma,^2, 4, 27^ and both inflammation and oxidative stress can cause neuronal cell death; therefore, we investigated whether blocking CXCR3 pathway would protect retinal neuronal cells from IOP-induced cell death. At 24 h after retinal ischemia, retinal cell apoptosis, as determined by measuring cytoplasmic histone-associated DNA fragmentation using a Cell death ELISA kit, was increased approximately eightfold in ischemia-injured WT retinas. This increase was reduced by 33% in retinas from mice lacking CXCR3 (Figure 3a). Further analysis of apoptotic cells by terminal deoxynucleotidyl transferase-mediated biotinylated UTP nick end labeling (TUNEL) assay revealed that TUNEL-positive (apoptotic) cells were mainly localized in neurons in the GCL and INL at 6 h after retinal ischemia. At 24 h after retinal ischemia, apoptotic cells were localized in the GCL, INL and, outer nuclear layer although were more prominent in the INL. Similarly, the number of apoptotic cells was significantly reduced in retinas from CXCR3 ko mice (Supplementary Figure S1). Consistent with alternations in cell apoptosis, H&E-stained retinal sections showed that the neuronal cells in the GCL were strikingly decreased in WT retinas at 7 days after ischemic injury. By contrast, GCL neurons were preserved in retinas of CXCR3 ko mice (Figure 3b). This result was further confirmed by counting neuron-specific nuclear protein-positive cells within the GCL in flat-mounted retinas by confocal image analysis at 7 days and 14 days after ischemic injury (Figure 3c and Supplementary Figure S2). Overall, these data demonstrated that the CXCR3 pathway is critically involved in ischemic injury-induced GCL neuronal death.

To test whether the activation of CXCR3 is sufficient to induce retinal inflammation and neuronal injury, we injected CXCL10 intravitreally and determined leukocytes recruitment and retinal cell apoptosis. As shown in Figure 3d, intravitreal delivery of CXCL10 elicited massive influx of leukocytes into the superficial retina in WT mice, similar to that occurred after retinal ischemic injury (Figure 2a). This effect was absent after deleting CXCR3, indicating specific CXCR3-mediated response. Associated with increases in retinal inflammatory reactions, CXCL10 treatment also induced cell death in WT retina but not in CXCR3-deficient retina (Figure 3e). Analysis of cell apoptosis by TUNEL assay revealed that CXCL10-induced apoptotic cells were localized in neurons in the GCL, INL and, outer nuclear layer (Supplementary Figure S3). Taken together, these results implicate the CXCR3 ligands/CXCR3 pathway as a key player in retinal inflammatory reactions and neuronal injury.

### Upregulation of CXCL10 and CXCR3 is medicated by ER stress

**Figure 4:**
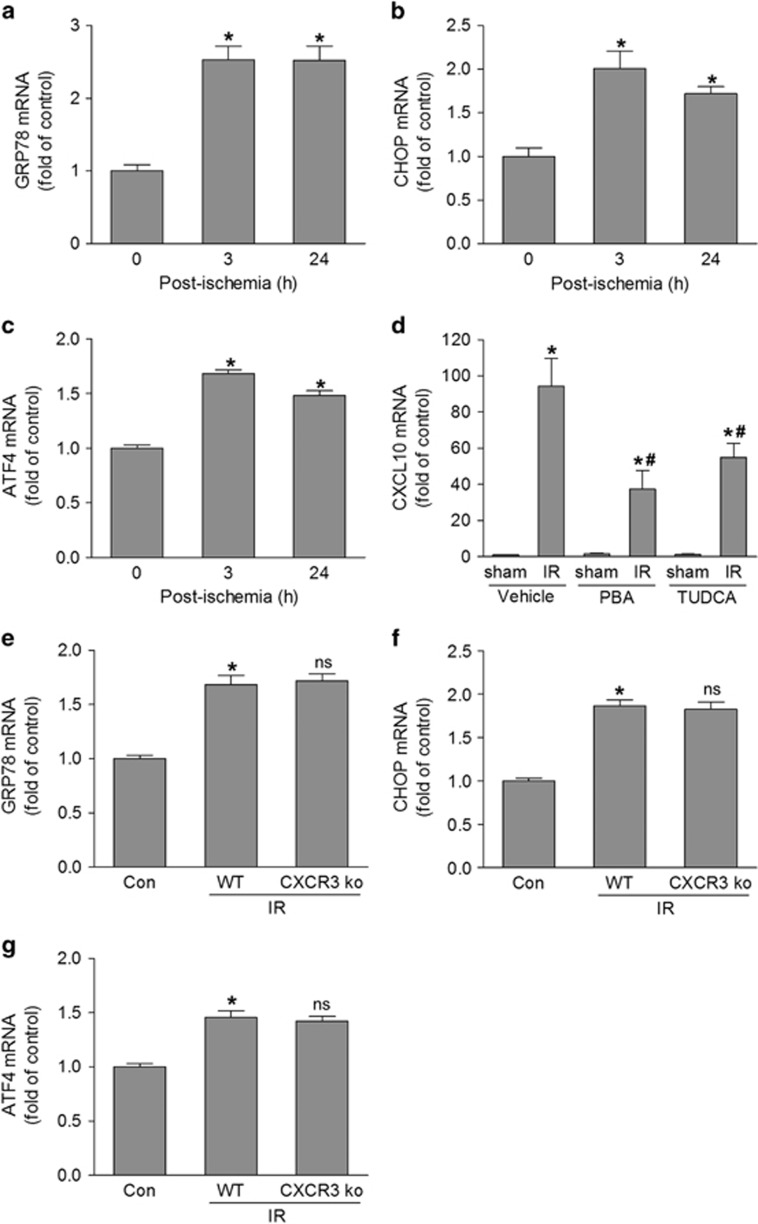
ER stress is involved in CXCL10 expression. (**a**–**c**) WT mice were exposed to IR and retinas were collected at 3 and 24 h after injury. The expression of GRP78, CHOP, and ATF4 mRNA was analyzed by qPCR. *P<0.05 compared with control. (**d**) WT mice were injected intraperitoneally with ER stress inhibitors, 4-phenylbutyric acid (PBA) or tauroursodeoxycholic acid (TUDCA) at 1 h before IR injury. Retinas were collected 6 h later and CXCL10 mRNA expression was assessed by qRT-PCR. *P<0.05 compared with sham control; ^#^*P*<0.05 compared with vehicle-treated IR. (**e**–**g**) Twenty-four hours after WT and CXCR3 ko mice were exposed to IR injury, retina mRNA was extracted to analyze ER stress target genes (GRP78, CHOP and ATF4) by qPCR. *P<0.05 *versus* control; ns: not significant compared with WT-IR. *N*=4

Given that CXCL10 and its receptor CXCR3 are involved in retinal inflammation and neuronal injury, it would be important to understand the potential mechanisms underlying the upregulation of this pathway after retinal ischemia. The ER represents the cellular quality control site for the folding and assembly of proteins. When the function of ER is perturbed, it causes ER stress, which helps to compensate for damage. However, it may trigger cell death if the ER stress is severe. Because ER stress has key roles in inflammation in many diseases and retinal neuronal injury,^28, 29, 30, 31^ we explored the potential role of ER stress in regulation of CXCL10/CXCR3 pathway. At 3 and 24 h after retinal ischemia, expression of ER stress markers (GRP78, CHOP, and ATF4) (Figures 4a–c) was significantly increased, indicating that ER stress was elevated in our model. To determine the role and mechanism of ER stress in CXCL10 expression, we then treated mice with 4-phenylbutyric acid and tauroursodeoxycholic acid, which are chemical chaperones that are widely used to block ER stress,^32, 33, 34^ and assessed CXCL10 expression. Inhibiting ER stress by both 4-phenylbutyric acid and tauroursodeoxycholic acid treatment attenuated CXCL10 expression by 61 and 43%, respectively, compared with untreated mice (Figure 4d). However, expression of ER stress markers were not altered by deleting CXCR3 (Figure 4e–g), suggesting that ER stress is not a downstream event after CXCR3 activation.

**Figure 5:**
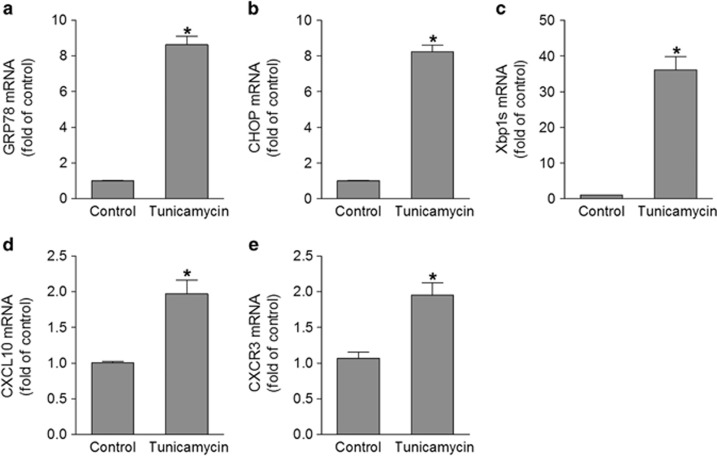
ER stress induction promotes CXCL10 expression. Primary retinal ganglion cells were isolated and treated with 2 μg/ml of tunicamycin for 24 h. The mRNA levels of ER stress target genes (GRP78, CHOP, and Xbp1s) (**a**–**c**), CXCL10 (**d**), and CXCR3 (**e**) were quantified by qPCR. *P<0.05 compared with control. *N*=4

We further determined whether induction of ER stress is sufficient to induce the upregulation of CXCL10 and CXCR3 pathway. As CXCL10 mRNA and the immunoreactivity of CXCR3 were positive in GCL neurons (Figures 1c and f), we cultured mouse primary retinal ganglion cells (RGCs) and treated them with the ER stress inducer tunicamycin. The expression of ER stress markers was markedly increased after tunicamycin treatment, demonstrating successful induction of ER stress. Accordant with the upregulation of ER stress (Figures 5a–c), expression of CXCL10 and CXCR3 was increased by approximately twofold (Figures 5d and e). These results indicate that ER stress serves as an upstream signaling pathway to upregulate CXCL10 and CXCR3 expression after ischemic injury.

### CXCR3 pathway is involved in ER stress-induced retinal cell death

**Figure 6:**
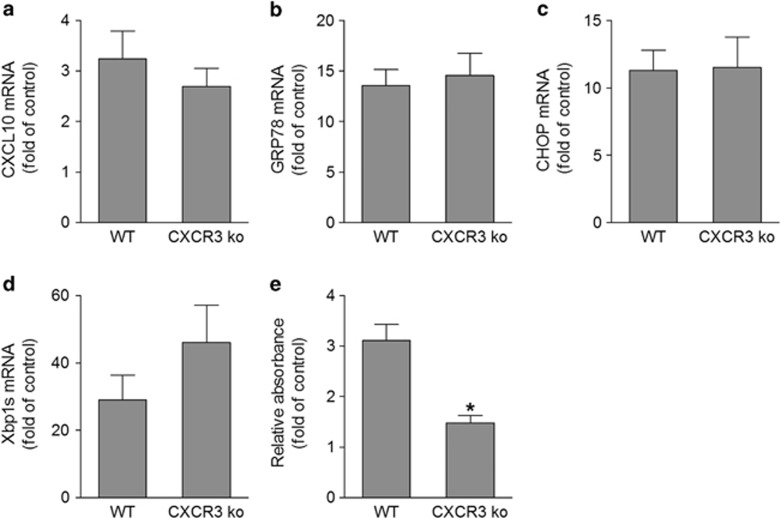
CXCR3 deletion attenuates ER stress-induced retinal cell apoptosis. WT and CXCR3 ko mice were intravitreally injected with tunicamycin (1 μg/eye) and retinas were collected at 24 h after injection. (**a**–**d**) qPCR was performed to measure the mRNA expression of CXCL10 and ER stress markers GRP78, CHOP, Xbp1s. (**e**) Apoptosis was analyzed by detecting histone-associated DNA fragmentation. *P<0.05 compared with tunicamycin-injected WT. *N*=3

As ER stress can induce expression of CXCL10 and CXCR3 and the activation of CXCR3 pathway is involved in retinal neuronal cell death (Figures 3, 4, 5), we investigated whether ER stress-induced CXCL10 expression has a role in ER stress-induced retinal neuronal injury. To do so, we injected tunicamycin intravitreally to WT and CXCR3-deficient mice. Tunicamycin induced significant increases in levels of CXCL10 and ER stress markers in retinas (Figures 6a–d) and there was no difference between WT and CXCR3-deficient mice, suggesting both genotypes respond to tunicamycin similarly. However, retinal cell apoptosis was induced threefold in retinas of WT mice compared with untreated controls, whereas it was only slightly induced in CXCR3-deficient mice (Figure 6e). These data support the notion that CXCR3 activation has an important role in ER stress-induced retinal neuronal injury.

## Discussion

During acute glaucoma, increased IOP leads to retinal neuronal injury, but the mechanisms remain to be defined. Here we provide the first evidence that activation of chemokine receptor CXCR3 is critically involved in retinal inflammation and neuronal cell damage. Using a mouse model of acute glaucoma, we found that IOP-induced retinal ischemic injury increases the expression of CXCR3 as well as its two ligands CXCL10 and CXCL4 in retina. By blocking CXCR3 with gene deletion or activating CXCR3 with intravitreal injection of CXCL10, we clearly demonstrate that CXCR3 is involved in retinal inflammation, oxidative, and nitrosative stress, and retinal neuronal death during acute glaucoma. This study, together with previous studies showing that activation of CXCR3 induces damage of trabecular tissue and consequently increases IOP and our ongoing work showing CXCL10 is increased in a mouse model of open-angle glaucoma (data not shown),^35^ highlights the potential value of CXCR3 as a target for glaucoma therapy, as blocking CXCR3 has dual beneficial effects of protecting retinal neurons and reducing IOP. The limitation of current study is that the mouse model used has different aspects from the human acute angle closure glaucoma such as the site of putative pressure-induced damage in glaucoma, the anatomy of the optic nerve of the mouse and the higher level but shorter duration of induced IOP.^4^ A number of CXCR3 antagonists are in clinical development for treating diseases such as arthritis and psoriasis,^36^ future evaluation of the effects of these antagonists on patients with acute glaucoma would validate the role of CXCR3 in human subjects and may lead to novel therapies.

The interaction of chemokines and their receptors have a key role in guiding leukocytes migration during inflammatory reactions. In spite of having been extensively studied in inflammatory diseases in other organs, the involvement of chemokines in ocular diseases has been less studied and most of these work has focused on severe ocular inflammatory conditions such as uveitis, infectious keratitis, allergic eye diseases, and corneal transplantation.^37^ In retinal diseases, the role of monocyte chemoattractant protein-1 and its receptor CCR2 are well known in age-related macular degeneration, diabetic retinopathy, and photoreceptor apoptosis after retinal detachment.^38, 39, 40, 41, 42^ In addition, CCR3 polymorphism is linked to age-related macular degeneration and blocking CCR3 inhibits choroidal neovascularization in a mouse model of wet age-related macular degeneration.^43, 44^ Our current finding that the CXCR3 pathway mediates retinal inflammation and neuronal injury in a model of acute glaucoma represents the first study that chemokines are involved in the pathogenesis of glaucomatous neuropathy. Given that chemokine receptors belong to the superfamily of G protein-coupled receptors, which are the major targets for drug development and numerous specific antagonists have been developed for chemokine receptors, further investigation of chemokines in acute and chronic inflammation during retinopathies will be clinically beneficial.

At present, the precise mechanisms by which activation of CXCR3 regulates retinal neuronal injury remain to be elucidated. In our study, CXCR3 ligands (e.g., CXCL10) are produced in the GCL cells of retina after ischemic injury. It is possible that these ligands bind to their receptor CXCR3, which is expressed in microglia, monocytes and activated T cells, and recruit them to the superficial retina. After activated by CXCL10 and/or other cytokine expressed in retina, these inflammatory cells produce more cytokines and chemokines to exaggerate inflammatory cascade, resulting in increase in leukocyte recruitment and progressive inflammation.^45, 46^ Some inflammatory cytokines produced by inflammatory cells further induce neuronal death by activating apoptotic signaling pathways.^47, 48, 49^ This possibility is supported by our data that deleting CXCR3 attenuates overall inflammatory reactions in ischemia-injured retina, including the reduction of microglia/monocyte recruitment/activation and the decreases in expression of adhesion molecule (E-selectin) and neural toxic and pro-inflammatory cytokine (interleukin-1*β*). In addition to neural toxic cytokines, reactive oxygen species and reactive nitrogen species generated by inflammatory cells also induce neuronal damage through a mechanism of oxidation and nitration in lipids and organelle proteins.^50^ Therefore, the reduction of oxidative stress and nitrosative stress by CXCR3 deletion also contributes to its neuroprotective effect. In line with this notion, deleting NOX2/NADPH oxidase, which is a major source of oxidative stress in inflammatory cells, similarly reduces peroxynitrite formation and protects neurons in the GCL.^27^ Overall, our data strongly suggest that CXCR3 is indirectly involved in retinal neuronal injury by recruiting inflammatory cells. However, CXCR3 signaling is reported to directly elicit apoptosis in fetal neurons.^51^ We demonstrate CXCR3 is markedly upregulated in GCL neurons after ischemic injury, thus we presume that CXCR3 might sensitize these neurons to CXCL10-induced apoptotic response. Further studies to specifically deleting CXCR3 in retinal neurons, microglia, and blood leukocytes are required to explore the precise mechanisms of CXCR3-induced retinal inflammation and neuronal injury in acute glaucoma. Regardless of the cell type-specific functions of CXCR3, blockade of CXCR3 with a pharmacological inhibitor may be beneficial for reducing retinal neuronal damage. Interestingly, loss of CXCR3 also prevents ER stress-induced retinal cell death, suggesting activation of the CXCR3 pathway is involved in neuronal injury in a variety of conditions and blocking this pathway is neuroprotective.

Although IbaI antibody stains both monocytes and microglia, by overlapping with GFP-positive cells, we found that IbaI-positive monocytes have different morphology from IbaI-positive microglia (Figure 2c). Infiltrated monocytes are smaller and have weaker immunoreactivity for anti-IbaI antibody. In addition, monocytes display a round or suborbicular morphology, whereas microglia exhibits a dendritic morphology. By comparing the morphology of IbaI-positive cells and their infiltration patterns in CXCR3-deficient retina with those in WT retina after ischemia-reperfusion (IR) (Figure 2a), our data show that CXCR3 deletion not only reduces microglial reactivity, but also prevents the massive infiltration of peripheral monocytes into superficial retina after IR. This result suggests that the CXCR3 pathway is critically involved in regulating monocyte recruitment during retinal inflammation.

Inflammation is involved in most neurodegenerative diseases, although the triggers of the inflammatory response are poorly characterized.^52^ CXCL10 is normally expressed at a low level under physiological conditions but is upregulated during infection or inflammation. In this study, we found that upregulation of CXCL10 is associated with increases in ER stress-related genes in ischemia-injured retina and blocking ER stress attenuates CXCL10 expression. On the other hand, the induction of ER stress promotes the expression of CXCL10 and its receptor. Although multiple cell types may express CXCL10 under stress, our study demonstrates that RGCs are one of the major sources of CXCL10 in response to ER stress. Moreover, CXCR3 expression is similarly upregulated by inducing ER stress in these cells. To our knowledge, this is the first report that ER stress regulates CXCL10 and CXCR3 levels under pathological condition. Although ER stress is able to activate NF-κB, IRF-1, and IRF-3, and these transcription factors regulate CXCL10 expression in response to different stimuli,^28, 53, 54, 55, 56^ the specific mediators linking ER stress to CXCL10 and CXCR3 expression in acute glaucoma remain to be elucidated.

In summary, our data reveal a novel mechanism that ER stress-regulated activation of CXCR3 pathway contributes to retinal inflammation and neuronal injury during acute glaucoma. As CXCL10 is also upregulated in other diseases associated with neuronal injury, such as diabetic retinopathy and stroke,^57, 58^ our study warrants further investigation of the CXCL10/CXCR3 axis in these diseases to determine whether or not this pathway has a general role in inducing inflammation and neuronal death and therefore targeting this pathway could be beneficial in a variety of diseases.

## Materials and Methods

### Animals

The experimental procedures and use of animals were performed in accordance with the Association of Research for Vision and Ophthalmology Statement for the Use of Animals in Ophthalmic and Vision Research, and all protocols were approved by the Institutional Animal Care and Use Committee at the University of Texas Medical Branch. C57BL/6 J, CXCR3KO and GFP transgenic mice were obtained from Jackson Laboratory (Bar Harbor, ME, USA) and maintained on a 12:12 light/dark cycle with food and water available *ad libitum*.

### Ischemia-reperfusion

Mice (8–12 weeks) were anesthetized with a mixture of ketamine hydrochloride (100 mg/kg) and xylazine hydrochloride (10 mg/kg) by intraperitoneal injection and 0.5% proparacine hydrochloride was administered topically. The anterior chamber of the right eye was cannulated with a 30-gauge infusion needle connected to a saline reservoir. IOP was raised to 110 mm Hg for 45 min by elevating the saline reservoir.^4, 27^ A sham procedure performed without elevating the pressure in the left eye served as the control. Eyes and retinas were collected from 3 h to 14 days after IR for further analysis. To investigate the involvement of ER stress in retinal pathogenesis, ER stress inhibitors, 4-phenylbutyric acid and tauroursodeoxycholic acid (Sigma-Aldrich, St Louis, MO, USA) were injected (i.p., 500 mg/kg) 1 h before IR was performed.

### Intravitreal injection

Intravitreal injection was performed as described previously.^59^ 1 μl PBS containing 1 μg of mouse CXCL10 (PeproTech, Rocky Hill, NJ, USA) was delivered by a 35-gauge needle. PBS was subjected as control to the contralateral eye of the same animal. After 24 h of injection, eyes and retinas were collected for further analysis.

### Bone marrow transplantation

Bone marrow transplantation was performed as described previous.^60^ In brief, WT mice at 6 weeks of age underwent whole-body gamma irradiation with 137Cs using a Gammacell 40 irradiator (MDS Nordion, Ontario, Canada). A lead shield was used to protect the head and eyes from radiation. A dose of 8.5 Gy (850 rads) was used to ablate the marrow. Within 24 h after irradiation, mice received bone marrow transplant from 6-week-old GFP transgenic mice through tail vein as a 200 *μ*l cell suspension containing 0.7–1.0 × 10^7^ cells. 6 weeks later, mice were subjected to IR.

### Isolation of primary RGC

Primary RGCs were isolated from WT mouse pups at postnatal day 3. Detailed procedures have been described in the previous study.^61, 62^ In brief, collected retinas were dissociated in a papain solution (15 U/ml) and incubated with anti-macrophage antiserum to remove the macrophages and microglial cells. Non-adherent cells were then incubated with mouse Thy-1.2 antibody (BD Biosciences, San Diego, CA, USA) to isolate ganglion cells. Cells were seeded at a density of 2.3 × 10^5^ cells/well and incubated at 37 °C. The purity of the RGCs was determined by staining with mouse Tuj1 antibody (BioLegend, San Diego, CA, USA), a specific RGC marker. To determine whether tunicamycin could induce the expression of CXCL10, cells were exposed to 2 μg/ml tunicamycin for 24 h.

### Real time quantitative RT-PCR analysis and primers used

Total RNA was isolated using RNAqueous-4PCR kit (Life Technologies, Carlsbad, CA, USA) according to the manufacturer's instruction. cDNA was generated by reverse transcription using High Capacity cDNA Reverse Transcription Kit (Life Technologies). Quantitative PCR was performed using a StepOnePlus PCR system (Life Technologies) with Power SYBR Green (Life Technologies). The fold difference in various transcripts was calculated by the ΔΔCT method using Hprt as the internal control. Primer sequences for mouse transcripts were as follows: Hprt For-5′-GAA AGA CTT GCT CGA GAT GTC ATG-3′ Hprt Rev-5′-CAC ACA GAG GGC CAC AAT GT-3′ CXCL10 For-5′-CAT CCC TGC GAG CCT ATC C-3′ CXCL10 Rev-5′-CAT CTC TGC TCA TCA TTC TTT TTC A-3′ CXCL4 For-5′-CGG TTC CCC AGC TCA TAG C-3′ CXCL4 Rev-5′-CCG GTC CAG GCA AAT TTT C-3′ CXCR3 For-5′-TTG CCC TCC CAG ATT TCA TC-3′ CXCR3 Rev-5′-TGG CAT TGA GGC GCT GAT-3′ GRP78 For-5′-ACT TGG GGA CCA CCT ATT CCT-3′ GRP78 Rev-5'-ATC GCC AAT CAG ACG CTC C-3′ ATF4 For-5′-TCC TGA ACA GCG AAG TGT TG-3′ ATF4 Rev-5′ACCCATGAGGTTTCAAGTGC-3′ CHOP For-5'-CTG GAA GCC TGG TAT GAG GAT-3′ CHOP Rev-5′-CAG GGT CAA GAG TAG TGA AGG T-3′ Xbp1s For-5′-TGC TGA GTC CGC AGC AGG TG-3′ Xbp1s Rev-5′- GCT GGC AGG CTC TGG GGA AG-3′.

### Western blot

Proteins were isolated from neuronal retina and subjected to SDS-PAGE. PVDF membranes, to which the proteins had been transferred, were incubated with primary antibody against nitrotyrosine (1 : 500; Cayman Chemical, Ann Arbor, MI, USA), followed with HRP-conjugated goat anti-mouse secondary antibody (1 : 2000; Amersham Biosciences, Piscataway, NJ, USA). Proteins were detected using the enhanced chemiluminescence system (Pierce, Rockford, IL, USA). The membranes were reprobed with monoclonal antibody against *β*-actin (1 : 5000; Sigma-Aldrich) as a loading control.

### *In Situ* hybridization

Retinal frozen sections were incubated with mouse CXCL10 probe (Advanced Cell Diagnostics, Hayward, CA, USA) and sequentially hybridized to a cascade of signal amplification molecules from RNAscope Fluorescent Multiplex detection reagent kit (Advanced Cell Diagnostics) according to the manufacturer's instructions. At last, slides were counterstained with DAPI to label nuclei and viewed by epifluorescence microscope to detect CXCL10 mRNA-positive cells.

### Immunohistochemical analysis

Retinal frozen sections were fixed in 4% paraformaldehyde in PBS, washed, and blocked with PowerBlock (Biogenx, San Ramon, CA, USA) for 1 h. Subsequently, sections were incubated with primary antibody against CXCR3 (1 : 250; R&D Systems, Minneapolis, MN, USA). After washing, retinal sections were incubated with Alexa Fluor 594-labeled goat anti-rabbit secondary antibody (1 : 1000; Life Technologies). Coverslips were mounted on slides with Fluoroshield with DAPI histology mounting medium (Sigma-Aldrich) and sections were examined by an Olympus 1X71 epifluorescence microscope.

### Immunostaining of retinal whole mounts

After the fixation in 4% paraformaldehyde, retinas were dissected from choroid and sclera, blocked and permeabilized in PBS containing 5% normal goat serum and 0.3% Triton-X-100 for 1 h. Subsequently, retinas were stained with Alexa Fluor 594-labeled isolectin B4 *(Griffonia simplicifolia)* (1 : 200; Life Technologies) and antibody against neuron-specific nuclear protein (1 : 400; Millipore, Billerica, MA, USA) or Iba1 (1 : 400; Wako, Osaka, Japan) overnight at 4 °C. Retinas were then incubated with Alexa Fluor 488-conjugated donkey anti-mouse or Alexa Fluor 488-labeled goat anti-rabbit secondary antibody (1:400). After washing with PBS, retinas were mounted on a microscope glass slide using Vectashield mounting medium (Vector Laboratories, Burlingame, CA, USA), and examined by confocal microscopy.

### ELISA

CXCL10 concentration in ischemia-reperfused retina was determined using Quantikine Mouse CXCL10 Immunoassay Kit (R&D Systems) following the manufacturer's instructions. The optical density was measured using Synergy H1 microplate reader (BioTek, Winooski, VT, USA).

### Cell death detection ELISA

To detect the apoptotic cell death in the ischemic injured retinas of WT or CXCR3 ko mice, a cell death detection ELISA^PLUS^ kit (Roche Molecular Biochemicals, Indianapolis, IN, USA) was used to quantify DNA fragmentation according to the manufacturer's instructions. The optical density was measured using Synergy H1 microplate reader.

### The TUNEL assay

To visualize apoptotic cells, TUNEL assay was performed on retinal frozen sections with ApopTag Fluorescein *in situ* Apoptosis Detection Kit (Millipore) according to the manufacturer's instructions. Retinal sections were counterstained with DAPI to label nuclei and TUNEL-positive cells were counted under epifluorescence microscope.

### Statistical analysis

Data were presented as Mmean±S.E.M. Results were analyzed using GraphPad Prism (GraphPad Software Inc., La Jolla, CA, USA). Comparison between experimental groups was made by Student's *t*-test and one-way ANOVA followed by *post hoc* Student's *t*-test using the Student–Newman–Keuls method. A value of *P*<0.05 was considered statistically significant. Data shown are representative of three or more independent experiments.

## Abbreviations

AMD: age-related macular degeneration
BM: bone marrow
CNS: central nervous system
ECL: enhanced chemiluminescence
ELISA: enzyme-linked immunosorbent assay
ER: endoplasmic reticulum
GCL: ganglion cell layer
GFP: green fluorescent protein
INL: inner nuclear layer
IOP: intraocular pressure
IR: ischemia-reperfusion
NeuN: neuron-specific nuclear protein
ONL: outer nuclear layer
PBA: 4-phenylbutyric acid
RGC: retinal ganglion cell
TUDCA: tauroursodeoxycholic acid
TUNEL: terminal deoxynucleotidyl transferase-mediated biotinylated UTP nick end labeling
WT: wild-type
