# Supplementary figures and images for: Endoplasmic reticulum stress-regulated CXCR3 pathway mediates inflammation and neuronal injury in acute glaucoma
Source: Cell Death Dis. 2015 Oct 8;6(10):e1900–. doi: 10.1038/cddis.2015.281 (PMC4632306; doi:10.1038/cddis.2015.281)

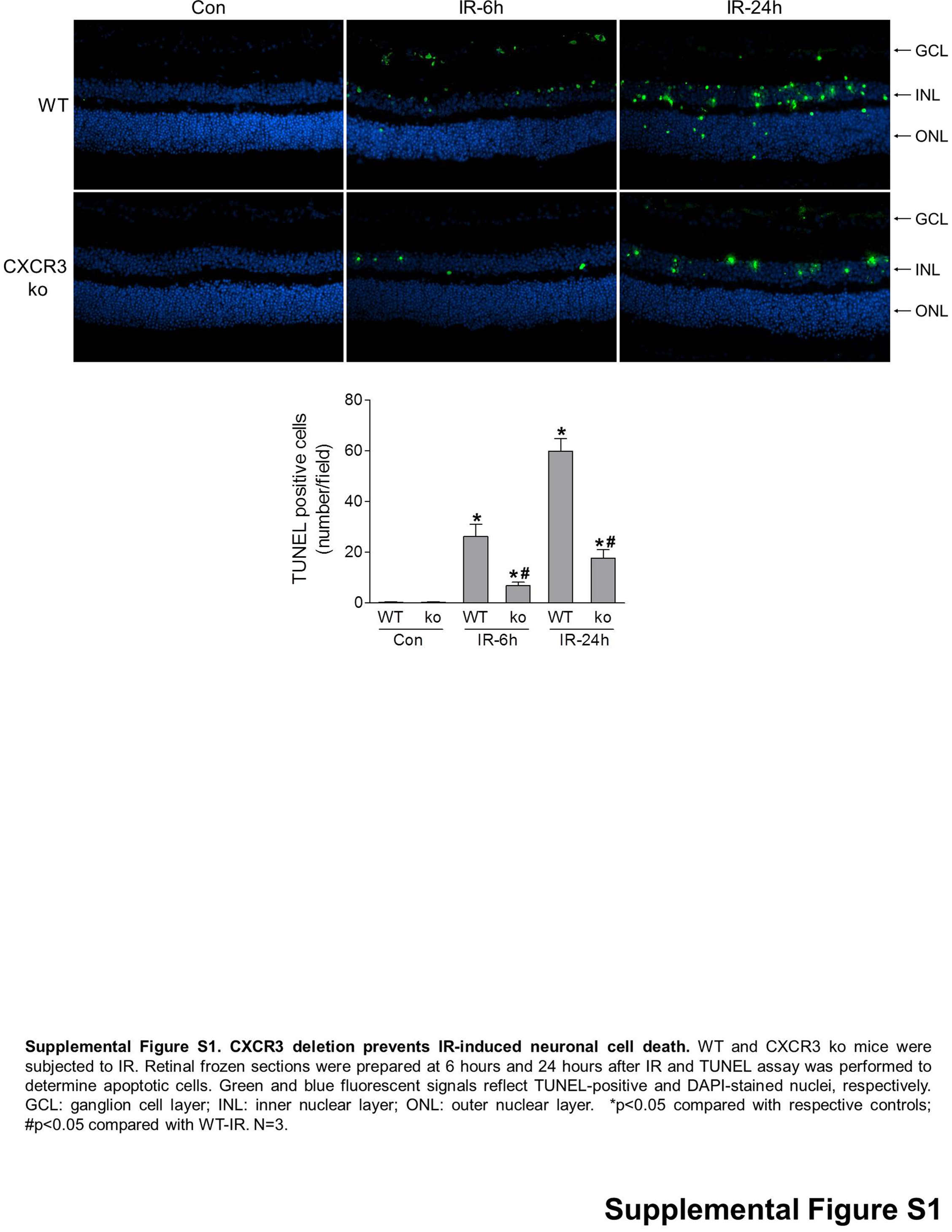

Supplement: Supplementary Figure 1 [file cddis2015281x1.tif]

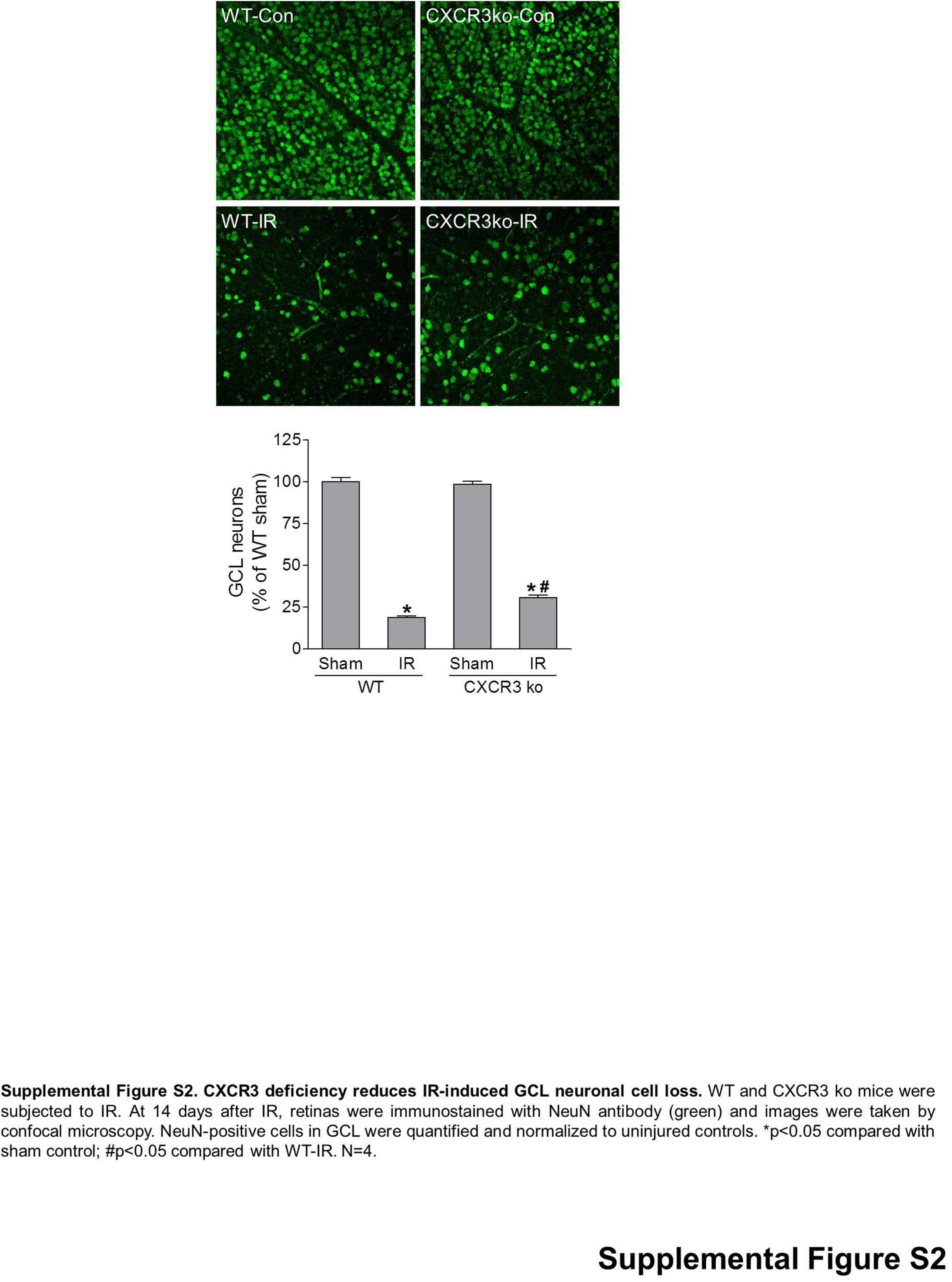

Supplement: Supplementary Figure 2 [file cddis2015281x2.tif]

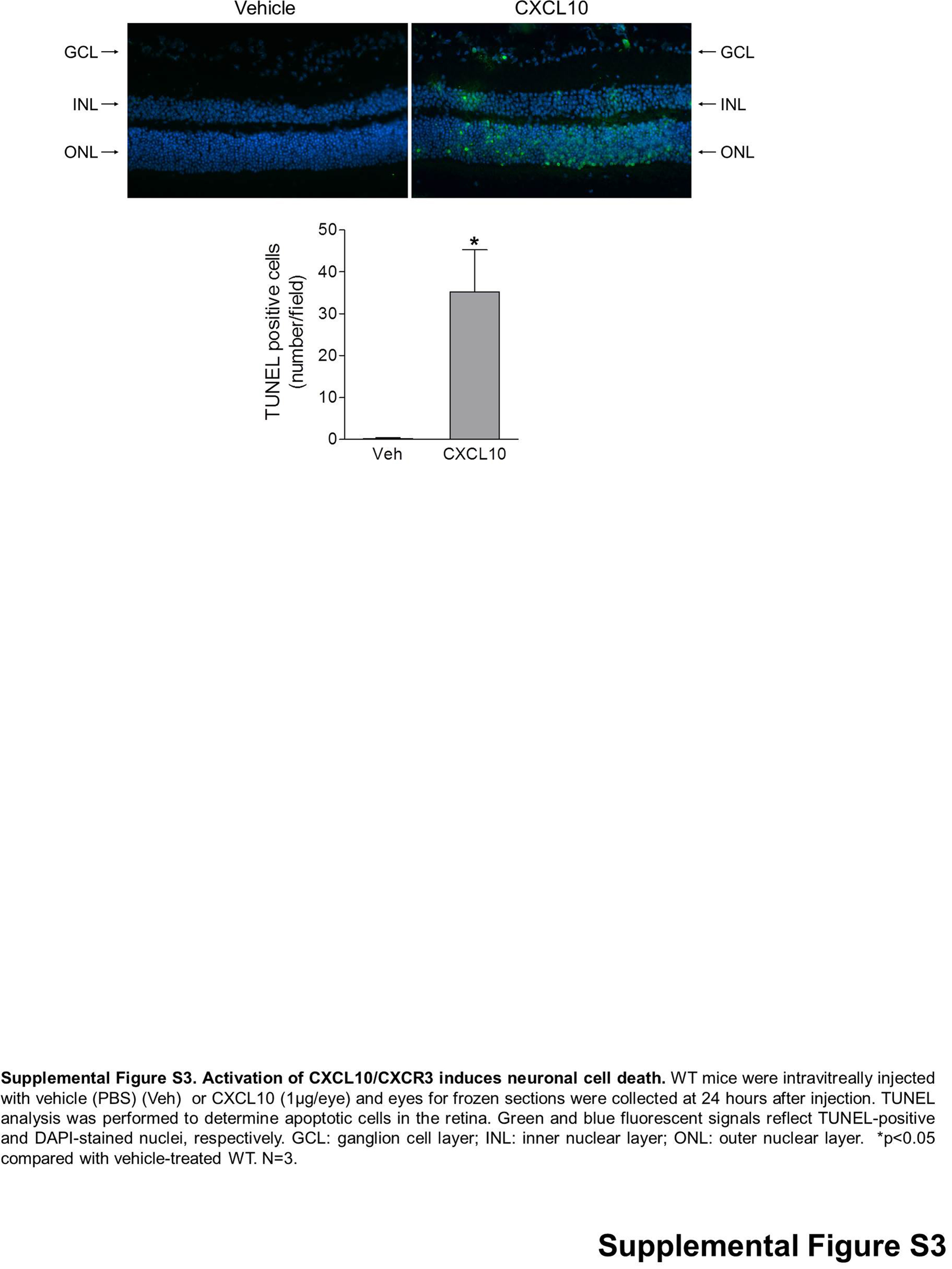

Supplement: Supplementary Figure 3 [file cddis2015281x3.tif]
